# Magnetic Nanoparticles Behavior in Biological Solutions; The Impact of Clustering Tendency on Sedimentation Velocity and Cell Uptake

**DOI:** 10.3390/ma13071644

**Published:** 2020-04-02

**Authors:** Mohammad Dabaghi, Ingrid Hilger

**Affiliations:** Institute of Diagnostic and Interventional Radiology, Experimental Radiology, Jena University Hospital-Friedrich Schiller University Jena, D-07747 Jena, Germany; mohammad.dabaghi@med.uni-jena.de

**Keywords:** magnetic nanoparticle, cell uptake, sedimentation and diffusion velocities, clustering

## Abstract

Magnetic nanoparticles (MNPs) are prone to exhibit physicochemical changes caused by their interaction with biological solutions. However, such interactions have been less considered in cancer therapy studies. The behavior of four iron oxide MNP formulations with different surface coatings, namely, chitosan (CS), polyvinyl alcohol (PVA), carboxymethyldextran (CMX), and polydimethylamine (PEA), was investigated, after their exposure to four different cell culture media (DMEM/F12 and MEM, among others) and six different cancer cell lines (HT29, HT1080, T24, MDA-MB-231, BxPC-3, and LS174T). The sedimentation (V_s_) and diffusion (V_d_) velocities of MNPs in different culture media were calculated. Atomic absorption spectroscopy (AAS) and dynamic light scattering (DLS) were used to quantify cell uptake efficiency and physicochemical properties, respectively. Apart from PVA-coated MNPs, CMX-, CS-, and PEA-coated MNPs clustered and increased notably in size when dispensed in culture media. The different MNP formulations led either to a low (PVA-coated MNPs), medium (CS- and CMX-coated MNPs), or high (PEA-coated MNPs) clustering in the different culture media. Clustering correlated with the V_s_ and V_d_ of the MNPs and their subsequent interaction with cells. In particular, the CMX-coated MNPs with higher V_s_ and lower V_d_ internalized more readily than the PVA-coated MNPs into the different cell lines. Hence, our results highlight key considerations to include when validating nanoparticles for future biomedical applications.

## 1. Introduction

Magnetic nanoparticles (MNPs), which are composed primarily of a magnetic core such as iron oxide and a polymeric surface coating, have interesting properties that can be exploited for many purposes. The broad spectrum of their applications in biomedical setups has recently aroused great interest. Various techniques for characterization of MNPs and utilization, advantages, and disadvantages of biomedical applications of MNPs, such as imaging, magnetic hyperthermia, and drug delivery, have recently been adequately discussed in a review article [1]. Many researchers have investigated nanoparticles for their applications in drug delivery [2,3,4,5,6,7] as well as diagnostic imaging and treatment [8,9,10,11]. MNPs with superparamagnetic magnetization have been widely used in disease imaging techniques such as magnetic resonance imaging (MRI) [12,13,14,15]. MNPs are capable of generating heat when exposed to an alternating magnetic field (AMF). This technique, known as hyperthermia, is usually used to kill tumor cells by raising the temperature up to 41–47 °C [16,17,18]. Several studies have presented promising results using the magnetic hyperthermia approach [4,5,19].

On the basis of the surface coating and reactive groups present, nanoparticles can be functionalized with various ligands or coupled to chemotherapeutics through covalent binding directly or with the aid of cross-linker molecules, electrostatically, or by encapsulation [5,20,21,22]. Hence, many researchers have employed nanoparticles to develop a drug delivery system for chemotherapeutic drugs in order to reduce the toxic side effects of the free drugs on healthy tissues and organs during treatment [4,23,24,25].

The applications of nanoparticles usually require preliminary investigations into their behavior in biological solutions. The uptake rate of nanoparticles by cancer cells is dependent on the stability and properties of the nanoparticles when exposed to biological solutions such as culture media or blood components such as serum proteins. The interactions between nanoparticles and molecular components of biological media cause nanoparticles to cluster or degrade, and thereby change in size, surface charge, and shape [26,27,28].

Interestingly, different MNP iron oxide cores may have almost the same properties (e.g., core material, size), yet the surface coating can lead to different behavior of those in the respective biological solutions used for validation. For example, defined molecules in solution can be adsorbed onto the nanoparticles in biological media and form a corona with varying compositions. It is reported that the corona changes the surface charge as it becomes negative in gold nanorods owing to a rapid adsorption of medium proteins [26]. In another study, gold nanoparticles introduced to plasma proteins caused a formation of the corona and an increase in the tendency of nanoparticles to cluster and their effective size [27]. Cho et al. also reported that the adsorption of serum proteins onto gold nanoparticles leads to an increase in size when dispersed in culture media, and this consequently increases the V_s_ and internalization into cells [29]. Mahmoudi and colleagues [30] reported different formation of protein corona on cetyltrimethylammonium bromide-stabilized gold nanorods after applying plasmonic heating, which might change the fate of nanoparticle in biological systems. Such alterations in the properties of nanoparticles have an impact on their therapeutic efficiency and should be considered in biomedical applications such as hyperthermia treatment. It is, therefore, important to investigate the less considered contribution of biological solutions to changes in the physicochemical parameters of MNPs and to study these changes in size, surface charge, shape, and so on, which in turn determine the clustering tendency and the V_s_ and V_d_ of MNPs. In addition, the role of the V_s_ and V_d_ of MNPs on cellular internalization still needs to be well researched, as the biomedical applications of MNPs, such as drug administration, hyperthermia treatment, and imaging modalities, are highly linked to their internalization rate in cells.

For further clarification, in the present study, we characterized chitosan (CS)-, polyvinyl alcohol (PVA)-, carboxymethyldextran (CMX)-, and polydimethylamine (PEA)-coated MNPs in detail to identify the best MNPs for further in vitro and in vivo therapeutic applications such as coupling with chemotherapeutic agents to develop a drug delivery system, imaging, application of hyperthermia treatment, and so on. Therefore, the behavior of these MNPs in terms of size, surface charge, clustering tendency, V_s_, and V_d_ in different culture media was investigated. Furthermore, the cellular uptake of two selected MNPs with different clustering behavior was validated on six different cancer cell lines. Finally, we compared the V_s_ and V_d_ of MNPs with their uptake levels in the cancer cell lines.

## 2. Materials and Methods 

### 2.1. MNPs and Chemicals

Different iron oxide MNPs (superparamagnetic) with varying surface coatings, chitosan (CS), polyvinyl alcohol (PVA), carboxymethyldextran (CMX), and polydimethylamine (PEA) in aqueous dispersion form were purchased from Chemicell GmbH (Berlin, Germany) (Table 1). The measured mean diameter of the MNP core was previously reported by our group in a publication with a diameter of approximately 12 nm [31]. Ammonium hydroxide solution was purchased from Sigma-Aldrich (St. Louis, MO, USA). Handling of the MNPs was according to the manufacturer’s instructions.

### 2.2. Cell Lines and Culture Media

Human colorectal adenocarcinomas (HT29, DSMZ GmbH, Braunschweig, Germany), human fibrosarcoma (HT1080, CLS GmbH, Eppelheim, Germany), and human bladder carcinoma (T24, DSMZ GmbH, Braunschweig, Germany) cells were cultured in DMEM/Ham’s F12 (1:1) with 10% fetal bovine serum (FBS). The human breast adenocarcinoma (MDA-MB-231, CLS GmbH, Eppelheim, Germany) cells were cultured in DMEM/Ham’s F12 (1:1) with 5% FBS, whereas the human pancreas adenocarcinoma (BxPC-3, ATCC, Manassas, VA, USA) cells were grown in RPMI 1640 with 10% FBS and 1 mM sodium-pyruvate. Human colorectal adenocarcinomas (LS174T, CLS GmbH, Eppelheim, Germany) cells were grown in MEM with 10% FBS, 1 mM sodium-pyruvate, and 0.1 mM non-essential amino acid (NEAA). All culture media were purchased from Gibco (Life Technologies Ltd, Paisley, UK). The varying components of each medium and their concentrations are categorized in Table 2. Cells were grown under standard conditions at 37 °C with 5% CO_2_ and 95% humidity.

### 2.3. Iron Content Measurement

In order to quantify the cell uptake of MNPs, cells were cultured with 100 µg Fe/mL MNPs in fresh medium at 37 °C for 24 h. Free MNPs (not internalized) were removed by washing the cells three times with Hank’s balanced salt solution, without Ca^2+^, Mg^2+^, and phenol red, and containing 0.35 g/L NaHCO_3_ (HBSS, Biochrom GmbH, Berlin, Germany). The cells were dissociated and counted using an automatic cell counter (Casy, Roche Innovatis AG, Reutlingen, Germany), and then subjected to determination of iron content by atomic absorption spectroscopy (AAS). Briefly, cell pellets were dissociated with extra pure 32% HCL (v/v, Carl Roth GmbH, Karlsruhe, Germany), by thorough mixing and incubation at room temperature for 30 min. Afterwards, 10% trichloroacetic acid (w/v, Carl Roth GmbH) was added and cells were centrifuged at 3720 g for 5 min to eliminate proteins from the suspension. The supernatant was used for iron determination on an AAS 5 FL spectrometer (Analytik Jena AG, Jena, Germany). Iron content per cell was calculated by normalizing the measured values to the total number of cells. The same procedure was used to quantify the iron content of MNPs through AAS.

### 2.4. Physicochemical Characterization of MNPs by Dynamic Light Scattering (DLS)

The hydrodynamic diameter, ζ-potential, and polydispersity index (PdI) of MNPs dispensed in water were measured by DLS using the Zetasizer Nano ZS (Malvern Instruments GmbH, Herrenberg, Germany). To validate the influence of different media on the physical properties of the MNPs, all four MNP formulations (PVA, CMX, CS, and PEA) were pre-treated with four different culture media listed in Table 2 for 24 h, and then subjected to magnetic separation prior to DLS. pH indicator paper (MACHEREY-NAGEL GmbH & Co. KG, Düren, Germany) was used to determine pH values. Iron concentration of MNPs was validated by AAS as described. In addition, to characterize the MNP´s core, cryo-transmission electron microscopy (TEM) of starch-coated fluidMAG-D MNP was performed, as described elsewhere [32]. Hereto, fluidMAG-D was used as a representative formulation for all MNPs used in this study. This procedure was valid as the core of all MNP formulations was made up by the utilization of the same standard protocol (information from the supplier). Briefly, an excess of liquid after applying the fluid MAG-D MNP dispersion to a copper grid covered with the holey carbon foil Quantifoil R3.5/1 (Micro Tools GmbH, Jena, Germany) was blotted between two filter paper strips. The samples were then frozen with cooled liquid ethane (to approximately −180 °C) in a cryobox (Carl Zeiss NTS GmbH, Oberkochen, Germany). A piece of filter paper was used to remove the additional ethane. The transfer of the samples into the pre-cooled cryo-transmission electron microscope Philips CM 120 (Philips Research, Eindhoven, The Netherlands) was performed with a cryo transfer unit (Gatan 626-DH) and images were acquired at 120 kV with a 1 K CCD camera FastScan F114 (TVIPS, Gauting, Germany).

### 2.5. Iron Staining and Microscopy (Prussian Blue)

To qualitatively validate the cellular uptake and localization of MNPs in cells as well as their behavior in the respective culture media by light microscopy, Prussian blue staining of iron was implemented. For this, 1 × 10^4^ cells were seeded in eight-well chamber slides (BD, Franklin Lakes, NJ, USA) and grown overnight under standard culture conditions. Thereafter, MNPs (100 µg Fe/mL) were added and the cells were further incubated for 24 h at 37 °C. Then, the cells were washed three times to get rid of free MNPs and fixed with 3.7% formaldehyde (Carl Roth GmbH & Co. KG, Karlsruhe, Germany) in phosphate-buffered saline (PBS). For staining, the slides were incubated in a solution consisting of equal volumes of 20% (v/v) HCl (Carl Roth GmbH & Co.) and 10% (w/v) potassium hexacyanoferrate (II) trihydrate (Sigma-Aldrich GmbH, Steinheim, Germany) for 30 min, and then rinsed with distilled water. The nuclei of the cells were subsequently stained with nuclear fast red aluminium sulphate solution (Carl Roth GmbH & Co.). The slides were separated from the chambers and the cells were mounted with Pertex (MEDITE GmbH, Burgdorf, Germany) and coverslipped for microscopy. Images were captured on an EVOS xl AMG microscope (PEQLAB, Erlangen, Germany) at 60× magnification.

### 2.6. Determination of the V_s_ and V_d_ of MNPs

*V_s_* of MNPs was calculated using the following equation [33].

(1) $$V_{s}=\frac{2g\left(p_{Fe}-p_{m}\right)d_{h}^{2}}{9\eta },$$

In Equation (1), *g* is the acceleration due to gravity; *p_Fe_* is the density of iron; *p_m_* is the density of culture medium; *d_h_* is the MNP’s hydrodynamic diameter in medium; and *η* is the viscosity of medium, which is 1.011 cP at 25 °C. 

Equation (2) was used to calculate *V_d_*.

(2) $$V_{d}=\left(\frac{x}{t}\right)=\frac{2D}{x}.$$

This equation is obtained according to [34], by modification of Einstein’s equation [35], *t = x^2^/2D*, in which *t* represents time, *x* is the distance of a particle’s diffusion, and *D* is the diffusion coefficient of the particle. 

The diffusion coefficient is calculated using the Stokes–Einstein equation (Equation (3)):

(3) $$D=\frac{k_{B}T\;}{6\pi \eta r},$$

where *k_B_* is defined as Boltzmann constant, *T* represents the absolute temperature, *η* is the viscosity of medium, and *r’* is the hydrodynamic radius (*d_h_*/2) of the MNPs. *V_d_* values are calculated for the MNPs when they have been considered to travel 1 mm or 10^−3^ m (x = 10^−3^).

As higher *V_s_* and lower *V_d_* result in faster settlement of a MNP dispensed in a solution, the V_s_/V_d_ ratio was calculated and used to obtain a better understanding of *V_s_* and *V_d_*.

### 2.7. Statistical Analysis

To evaluate the significance of the differences between cell uptake and *V_s_* and *V_d_* of MNPs, one-way analysis of variance (ANOVA) and independent samples *t*-test were used. *p* < 0.05 was considered statistically significant for the difference.

## 3. Results

### 3.1. Physicochemical Features of MNPs

The MNP formulations (same core, but different coatings) revealed almost comparable pH and morphological features (size, polydispersity, and so on). However, the ζ-potential was different for the MNPs. According to the supplier’s datasheet, each coating of MNPs has a specific functional group, as CS and PEA have amine groups (–NH_2_^−^), CMX has carboxyl groups electrostatically bound to a sodium ion (–COO–Na^+^), and PVA has hydroxy groups (–OH). These functional groups interact with H^+^ and OH^−^ ions in water. TEM was used to image fluidMAG-D MNP as a representative for the MNPs in this study. As TEM does not detect the MNP coating, but rather the iron oxide core of MNP, visualizing the fluidMAG-D MNP (Figure S1), this MNP formulation was considered a representative of the other ones, because its core is identical to those of CS-, PVA-, CMX-, and PEA-coated MNPs. The iron content of MNPs, measured by the AAS method, also appeared to be comparable (Table 3).

### 3.2. Behavior of MNPs in Different Culture Media

The characterization of the MNP formulations dispensed in four different culture media showed changes in the hydrodynamic diameter of all MNPs, which varied depending on the MNP coating composition (Table 4). Statistical analysis showed that the average size growth of PEA-coated MNPs was significantly higher than that of others (*p* < 0.01 compared with PVA-coated MNPs and *p* < 0.05 compared with CMX- and CS-coated MNPs). In contrast, PVA-coated MNPs’ average size growth in media was significantly lower than that of the others (*p* < 0.01). On the basis of the average size increase of the MNPs in different media, PVA-, CMX-, CS-, and PEA-coated MNPs were considered as low, medium, and high clustering MNPs, respectively. Using four different culture media to study the behavior of MNPs demonstrated that, according to the type of the cell culture medium, MNPs can behave differently. For instance, PVA-coated MNPs behaved the same in all four media, whereas the hydrodynamic diameter of the other three MNP formulations increased remarkably from moderate to high. On the other hand, the ζ-potential of PVA-, CS-, and PEA-coated MNPs turned negative, while it did not change remarkably for CMX-coated MNPs in all four media. This shows that, although the culture medium highly influences the behavior of MNPs, the properties of the MNPs themselves play a role in their characteristic changes in different media.

### 3.3. V_s_ and V_d_ of MNPs Dispensed in Different Cell Culture Media

On average, V_s_ of PVA-coated MNPs was significantly lower than CMX- (*p* < 0.05), CS- (*p* < 0.05), and PEA-coated MNPs (*p* < 0.05). Furthermore, V_s_ of the PVA-coated MNPs was independent of the culture media. On the other hand, V_s_ of PEA-coated MNPs was considerably higher than that of CMX- and CS-coated MNPs, although the differences were not significant (*p* = 0.054 and *p* = 0.052, respectively). The V_s_ of CMX- and CS-coated MNPs were also not significantly different. In addition, CS-, CMX-, and PEA-coated MNPs showed higher V_s_ when dispensed in culture medium 3 (Table 5).

### 3.4. Comparing the Impact of the V_s_/V_d_ Ratio of PVA-Coated MNPs (Low Clustering Behavior) and V_s_/V_d_ Ratio of CMX-Coated MNPs (High Clustering Behavior) on Cellular Uptake in Different Cancer Cell Lines

The comparison of V_s_ and V_d_ of PVA- and CMX-coated MNPs in different culture media with their cell uptake by different cell lines revealed the following: (a) CMX-coated MNPs generally sediment faster and defuse slower than PVA-coated MNPs in all six culture media (V_s_/V_d_ ratio for all culture media higher for medium clustering CMX-coated MNPs than for low clustering PVA-coated MNPs (*p* < 0.01), Table 6). (b) The sedimentation behavior of PVA-coated MNP in different media was almost similar (similar V_s_/V_d_ ratios). (c) The sedimentation behavior of CMX-coated MNP varied among the different cell culture media and did not always correlate with MNP cell uptake. Microscopic observations of six different cell lines incubated with PVA- and CMX-coated MNPs visualized the internalization of MNPs in cells (Prussian blue staining method). AAS substantiated the iron concentration of the cells after incubation with PVA- and CMX-coated MNPs. In line with the observations of microscopic images, Supplemental Figure S2a shows that MDA-MB-231, T24, and HT1080 cells internalized a significantly higher amount of PVA-coated MNPs than BxPC-3, HT29, and LS174T cells (24 h, *p* < 0.001 for MDA-MB-231, T24, and HT1080). Conversely, very low MNP internalization by BxPC-3, HT29, and LS174T cells could be detected (Supplemental Figure S2b). The experiments with cells incubated with CMX-coated MNPs, also in line with the observations of microscopic images (Supplemental Figure S3a), indicated that LS174T cells take up CMX-coated MNPs significantly lower (*p* < 0.001), but MDA-MB-231 cells internalize them significantly more readily than other cell lines tested (*p* < 0.001). There was no significant difference in the uptake of CMX-coated MNPs between BxPC-3, HT1080, T24, and HT29 cell lines (Supplemental Figure S3b). Comparing these results with the results for PVA-coated MNPs, we can say that, although MNPs internalize differently depending on their characteristics such as V_s_, V_d_, size, and coating, cell lines also behave differently and play an obvious role in the uptake rate of MNPs. However, in general, cells intended to significantly internalize more CMX-coated MNPs than PVA-coated MNPs (*p* < 0.001 for LS174T, HT29, BxPC-3, and MDA-MB-231 cells; *p* < 0.05 for T24 cells; and *p* < 0.05 for HT1080 cells).

## 4. Discussion

In the present study, we measured the size, ζ-potential, and pH of four MNPs coated with CS, PVA, CMX, and PEA, when dispersed in water. Although the size and pH of the MNPs were almost comparable to each other, the ζ-potential was different. We believe that the difference between the surface charges of the MNPs relates to the molecular structure and functional groups of the coating formulations. The interactions of these functional groups with H^+^ and OH^−^ ions in water determine the surface charge of MNPs.

Our findings concerning the clustering behavior of MNPs in various culture media revealed that all MNPs used in this study increased their size to different degrees and seemed to form clusters. It is known that, generally, greater van der Waals attractive forces than electrostatic repulsive forces between nanoparticles lead them to agglomeration and forming clusters [36]. The increase in the size of MNPs in different culture media was, however, not similar for all MNPs. Compared with others, the average size increase was significantly higher for PEA-coated MNPs in all culture media, while it was significantly lower for PVA-coated MNPs. Furthermore, the size of CMX-, CS-, and PEA-coated MNPs altered in different culture media, whereas the size of PVA-coated MNPs was nearly stable. We believe that the different clustering tendency of MNPs in this study is associated with several parameters such as the coating formulation and the culture medium components, particularly proteins. Nanoparticle-protein corona, which is defined as adsorption of proteins in biological media to the surface of nanoparticles, is reported in literature to affect the size and the surface properties of nanoparticles [37]. In our study, apart from the CMX-coated MNP, which was negatively charged when dispersed in water, the ζ potential of the MNPs in different culture media changed, as it became more negative for PVA-, CS-, and PEA-coated MNPs. This also indicates the adsorption of the proteins onto the surface of MNPs. In line with this relationship, Schäffler and co-workers [38] reported that the ζ-potential of gold spheres decreases owing to serum protein corona formation.

Following characterization of the MNPs in different culture media, we calculated their V_s_ and V_d_. The results presented in Table 5 show that PEA-coated MNPs sediment considerably faster than PVA-, CMX-, and CS-coated MNPs (*p* < 0.05, *p* = 0.054, and *p* = 0.052, respectively) and PVA-coated MNPs sediment significantly slower than the others (*p* < 0.05 for all). Furthermore, V_s_ of PVA-coated MNPs was independent of the culture media, whereas CS-, CMX-, and PEA-coated MNPs sediment faster in culture medium 3. According to the formula, V_s_ and V_d_ of MNPs with similar core, suspended in solutions with the same viscosity, mainly depend on the size of the nanoparticles. This underlines the fact that clustering behavior has the greatest influence on the V_s_ and V_d_ of MNPs. In line with that, we showed that CS-, CMX-, and PEA-coated MNPs form larger clusters in culture medium 3, where they sediment correspondingly faster.

To investigate how different clustering behavior might influence the cytotoxicity and cell uptake of MNPs, we incubated six different cell lines with PVA (low clustering) and CMX (medium clustering). Microscopic images proved the presence of CMX-coated MNPs inside the cells and, in some cases, adhered to the bottom of the flask (Supplemental Figure S3a). In contrast, it was challenging to find PVA-coated MNPs neither internalized to the cells nor adhered to the flask bottom (Supplemental Figure S2a). The quantification of the MNPs cell uptake also showed that all cell lines internalized more CMX-coated MNPs than PVA-coated MNPs (Supplemental Figures S2b and S3b). The uptake amount of CMX- or PVA-coated MNPs, however, was different among cell lines. In the case of CMX-coated MNPs, we found that low V_s_ is correlated with a low uptake. The CMX-coated MNPs were taken up by the LS174T cells at the lowest rate, whereby also the V_s_ was the lowest. Conversely, the highest uptake of CMX-coated MNPs corresponded to high V_s_ of these MNPs when incubated with MDA-MB-231 cells (11.2 ± 0.2 pg/cell). However, CMX-coated MNPs showed greater V_s_ when incubated with BxPC-3 cells. This indicates that CMX-coated MNPs are more likely to internalize in MDA-MB-231 cells than BxPC-3, underlining the importance of the cell uptake potential. We also observed that PVA was internalized unevenly by different cell lines, although the V_s_ were approximately similar when suspended in different culture media. For instance, HT1080, MDA-MB-231, and most notably T24 cell lines internalized significantly more PVA-coated MNPs than other cell lines, whereas their V_s_ were comparable. Such evidence reflects the role of cell uptake potential in the internalization of MNPs. Literature has also reported a variation in cell line uptake potential [39]. Several cellular features are able to determine the cell uptake potential. The cell proliferation rate can essentially affect the tendency of cells to internalize many components in the surrounding media such as MNPs. In addition, the mechanism and rate of endocytosis and exocytosis of MNPs that cells employ also differ from cell type to cell type.

Our data indicate that the functional groups and chemical structure of MNP’s coating determine the surface charge in water. The chemical structure of the coating is also likely to govern the adsorption of proteins onto the surface of MNPs, as we measured a varying increase in size among the MNPs in different culture media. For instance, the size of PVA-coated MNPs did not increase after introducing to the culture media and seemed to behave more individually or in clusters with few domains. However, CMX-, CS-, and PEA- coated MNPs clustered to a different extent when dispersed in the culture media. Obviously, the behavior of MNP to form larger clusters is related to the interaction of MNP surface matrix chemistry, charge, and functional groups of the matrix with the components of the culture media, as all the MNPs share the same core and have a comparable size in water. Interestingly, the clustering tendency resulting in a size increase, as a consequence of the interaction between the MNPs’ coatings and the components of the culture media, affects the Vs of MNPs. Namely, the V_s_ of the MNPs were different depending on their size in the culture media. Comparing the cell uptake of PVA-coated MNP as a low clustering MNP formulation and CMX-coated MNP as a medium clustering MNP formulation by different cell lines, we demonstrated that different cell lines are significantly more likely to take up CMX-coated MNPs. On the basis of Brownian motion, it is presumed that MNPs are evenly distributed in the culture medium and cells receive the same concentration as when they were initially added. However, the size increase of MNPs, as a result of clustering, which causes higher V_s_, might very well directly affect the dosage of MNPs to which cells are exposed. This explains the higher internalization of CMX-coated MNPs than PVA-coated MNPs, as CMX-coated MNPs interact more with cells owing to higher V_s_.

Taken together, we show that the surface coating and the immediate biological milieu influence the physicochemical properties of the MNPs, and consequently alter their clustering and V_s_ ability. This, in turn, impacts the MNP uptake behavior in vitro, which might be different according to the membrane properties of the respective cell line. In conclusion, the results of the present study highlight the great impact of the interaction between the surface coating matrix of the MNPs with biological components on the clustering of MNPs, and consequently on MNPs’ sedimentation and interaction with cells. Therefore, we strongly recommend that the clustering behavior should be investigated in advance (i.e., prior to in vitro and in vivo studies) in order to improve the understanding of MNPs’ capabilities for the intended biomedical application.

## Figures and Tables

**Table 1:** Supplier’s reported properties of the magnetic nanoparticles (MNPs) (size, coating compound, and weight of volume) used in this study. CS, chitosan; PVA, polyvinyl alcohol; CMX, carboxymethyldextran; PEA, polydimethylamine.

MNPs	Coating	Size	Weight of Volume (NP/mL)
fluidMAG-CS	Chitosan	100 nm	25 mg NP/mL
fluidMAG-PVA	Polyvinyl alcohol	100 nm	25 mg NP/mL
fluidMAG-CMX	Carboxymethyldextran	100 nm	25 mg NP/mL
fluidMAG-PEA	Polydimethylamine	100 nm	25 mg NP/mL

**Table 2:** Overview of culture media used in the study and their respective components and concentrations. FBS, fetal bovine serum; NEAA, non-essential amino acid.

Medium Components	Medium 1: DMEM/F12 + 10% FBS	Medium 2: DMEM/F12 + 5% FBS	Medium 3: RPMI 1640 + 10% FBS	Medium 4: MEM + 10% FBS
FBS/medium (v/v)	10%	5%	10%	10%
Amino Acids (mg/L)	1110	1110	994	848
Vitamins (mg/L)	34	34	44	8
Inorganic Salts (mg/L)	10,074	10,074	9349	10,022
D-Glucose (mg/L)	3151	3151	2000	1000
Sodium Pyruvate (mg/L)	55	55	0	0
Added Sodium Pyruvate (mM)	0	0	1	1
NEAA (mM)	0	0	0	0.1

**Table 3:** Physicochemical features of MNPs suspended in water. Properties of MNPs determined via dynamic light scattering (DLS), using pH indicator papers and atomic absorption spectroscopy (AAS). Mean and standard deviation of the mean (n = 3). PdI, polydispersity index.

MNPs	Size (d.nm)	PdI	ζ-Potential	pH	Iron Content (mg Fe/mL)
**CS-coated MNPs**	92.1 ± 0.4	0.11	27.4 ± 7.0	7	1.01 ± 0.13
**PVA-coated MNPs**	130.6 ± 2.1	0.10	4 ± 0.1	7	1.19 ± 0.11
**CMX-coated MNPs**	110.6 ± 3.5	0.07	–32.3 ± 0.1	7	0.98 ± 0.11
**PEA-coated MNPs**	116.5 ± 1.0	0.16	36.3 ± 0.3	7	1.05 ± 0.07

**Table 4:** Physicochemical properties of PVA-, CMX-, CS-, and PEA-coated MNPs suspended in four different culture media. See Table 2 for the components and concentrations of the media. Mean and standard deviation of the mean (n = 3).

MNPs	Parameter	Medium 1	Medium 2	Medium 3	Medium 4
**PVA-coated MNPs**	**Size (d.nm)**	148 ± 1	150 ± 2	144 ± 3	149 ± 1
	**PdI**	0.097 ± 0	0.104 ± 0	0.092 ± 0	0.096 ± 0
	**ζ-potential**	−17 ± 1	−15 ± 1	−15 ± 1	−20 ± 1
**CMX-coated MNPs**	**Size (d.nm)**	288 ± 1	356 ± 14	365 ± 12	263 ± 11
	**PdI**	0.197 ± 0	0.222 ± 0	0.268 ± 0	0.207 ± 0
	**ζ-potential**	−28 ± 0	−31 ± 0	−27 ± 0	−28 ± 1
**CS-coated MNPs**	**Size (d.nm)**	284 ± 13	290 ± 15	365 ± 33	264 ± 12
	**PdI**	0.164 ± 0	0.171 ± 0	0.2 ± 0	0.169 ± 0
	**ζ-potential**	−31 ± 1	−32 ± 1	−30 ± 0	−28 ± 0
**PEA-coated MNPs**	**Size (d.nm)**	784 ± 83	950 ± 34	1438 ± 218	630 ± 45
	**PdI**	0.293 ± 0	0.347 ± 0	0.481 ± 0	0.288 ± 0
	**ζ-potential**	−31 ± 1	−31 ± 0	−30 ± 0	−28 ± 1

**Table 5:** V_s_ and V_d_ as well as the size of PVA-, CMX-, CS-, and PEA-coated MNPs in different culture media. See Table 2 for the components and concentrations of the media. Mean and standard deviation of the mean of MNPs size in media (n = 3).

MNPs	Media	MNPs Size in Medium (d.nm)	Size Increase in Media	V_s_ (× 10^−10^ m/s)	V_d_ (× 10^−12^ m/s)	V_s_/V_d_
**PVA-coated MNPs**	1	148 ± 1	13%	3.30	6.07	0.54
	2	150 ± 2	15%	3.39	5.99	0.56
	3	144 ± 3	10%	3.12	6.24	0.5
	4	149 ± 1	14%	3.34	6.03	0.49
**CMX-coated MNPs**	1	288 ± 1	160%	12.51	3.12	4.01
	2	356 ± 14	222%	19.11	2.52	7.58
	3	365 ± 12	230%	20.09	2.46	8.17
	4	263 ± 11	138%	10.43	3.41	3.06
**CS-coated MNPs**	1	284 ± 13	208%	12.14	3.16	3.84
	2	290 ± 15	215%	12.66	3.10	4.08
	3	365 ± 33	296%	20.05	2.46	8.15
	4	264 ± 12	187%	10.54	3.40	3.1
**PEA-coated MNPs**	1	784 ± 83	573%	92.63	1.14	81
	2	950 ± 34	715%	136.1	0.94	144
	3	1438 ± 218	1134%	312.1	0. 62	500
	4	630 ± 45	441%	59.89	1.42	42

**Table 6:** Comparing the influence of V_s_ on cell uptake of low and medium clustering MNPs (PVA-coated MNPs vs. CMX-coated MNPs). V_s_ and V_d_ are reported in meter per second (m/s). The uptake amount is reported in picogram iron per cell (pg Fe/cell). Four different cell culture media were used in this study (defined in the material and method section). HT1080, T24, and HT29 cell lines were cultured in culture medium 1, MDA-MB-231 in culture medium 2, BxPC-3 in culture medium 3, and LS174T in culture medium 4.

MNPs	Cell Lines	Media	V_s_ (× 10^−10^ m/s)	V_d_ (× 10^−12^ m/s)	V_s_/V_d_	Uptake of MNPs (pg Fe/cell)
**PVA-coated MNPs**	**HT1080**	1	3.30	6.07	0.54	1.0 ± 0.0
	**T24**	1	3.30	6.07	0.54	3.7 ± 0.3
	**HT29**	1	3.30	6.07	0.54	0.1 ± 0.0
	**MDA-MB-231**	2	3.39	5.99	0.56	1.9 ± 0.2
	**BxPC-3**	3	3.12	6.24	0.5	0.2 ± 0.0
	**LS174T**	4	3.34	6.03	0.49	0.2 ± 0.0
**CMX-coated MNPs**	**HT1080**	1	12.51	3.12	4.01	4.8 ± 1.4
	**T24**	1	12.51	3.12	4.01	4.6 ± 0.1
	**HT29**	1	12.51	3.12	4.01	4.8 ± 0.2
	**MDA-MB-231**	2	19.11	2.52	7.58	11.2 ± 0.2
	**BxPC-3**	3	20.09	2.46	8.17	4.6 ± 0.1
	**LS174T**	4	10.43	3.41	3.06	1.3 ± 0.0

## Supplementary Materials

The following are available online at https://www.mdpi.com/1996-1944/13/7/1644/s1, Figure S1: TEM image of the iron oxide core of fluidMAG-D MNPs; Figure S2: Internalization of PVA-coated MNPs in six different cell lines; Figure S3: Internalization of CMX-coated MNPs in six different cell lines.
