# Supplementary material for: Magnetic Nanoparticles Behavior in Biological Solutions; The Impact of Clustering Tendency on Sedimentation Velocity and Cell Uptake
Source: Materials (Basel). 2020 Apr 2;13(7):1644. doi: 10.3390/ma13071644 (PMC7178374; doi:10.3390/ma13071644)
Supplement: Supplementary file 1 [file materials-13-01644-s001.pdf]

materials

Supplementary Materials

Magnetic Nanoparticles Behavior in Biological Solutions; The Impact of Clustering Tendency on Sedimentation Velocity and Cell Uptake

Mohammad Dabaghi and Ingrid Hilger *

Institute of Diagnostic and Interventional Radiology, Experimental Radiology, Jena University Hospital-Friedrich Schiller University Jena, D-07747 Jena, Germany
* Correspondence: ingrid.hilger@med.uni-jena.de; Tel.: +49-3641-932592

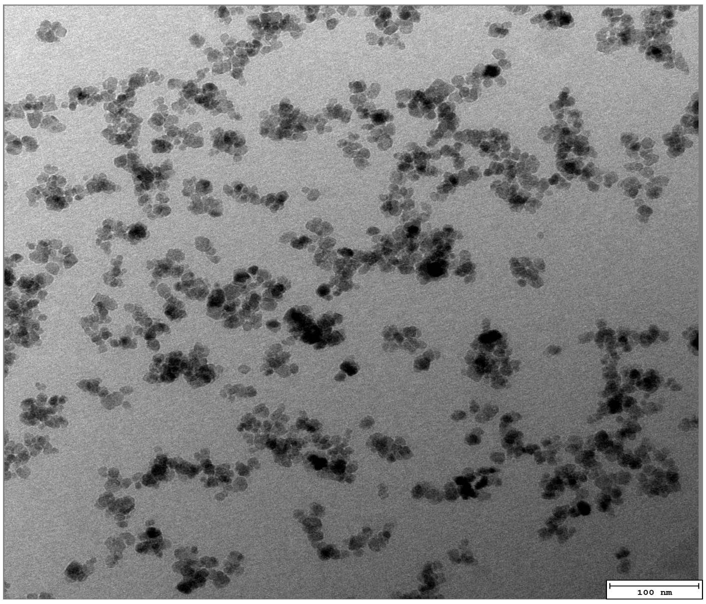

Figure S1. Representative TEM image of the iron oxide core of fluidMAG-D MNPs. The fluidMAG-D MNP TEM image is representative of all the other MNPs with an approximate core diameter of 10-15 nm, which is identical to the core of CS-, PVA-, CMX- and PVA-coated MNPs. The image displays only the iron oxide cores of the MNPs. The coating is not visible.

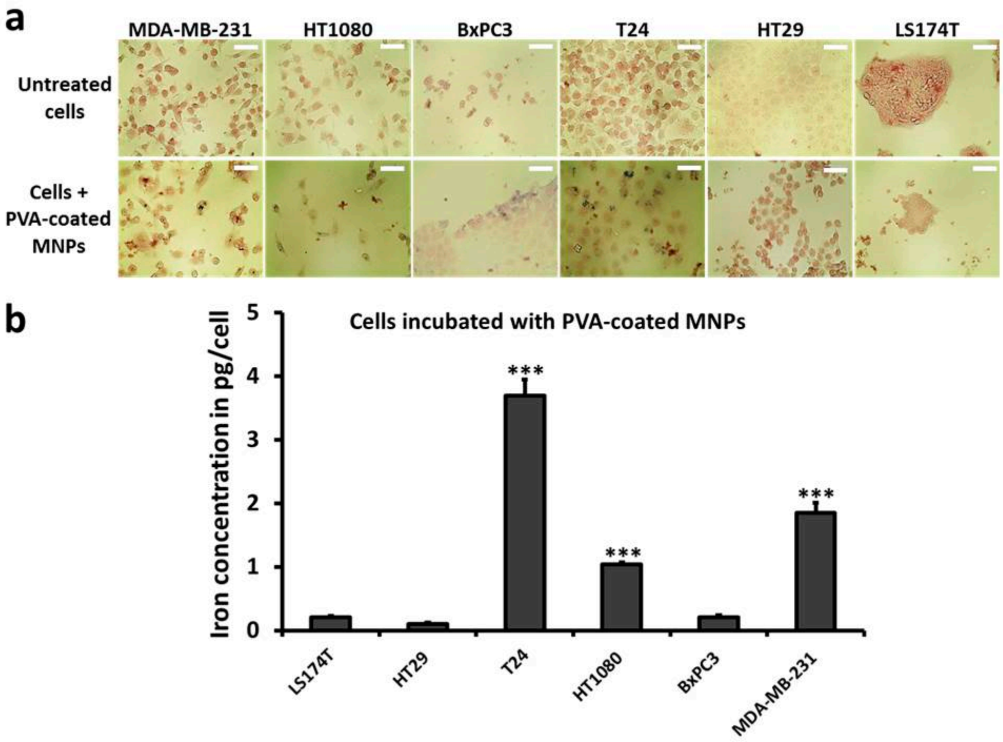

Figure S2. Internalization of PVA-coated MNPs in six different cell lines. Microscopic images of MDA-MB-231, HT1080, BxPC-3, T24, HT29 and LS174T cells internalizing (low clustering) PVA-coated MNPs (**a**) and the quantified uptake amount of PVA-coated MNPs measured by AAS (**b**). Six different cell lines were incubated for 24 h with 100 µg Fe/mL PVA-coated MNPs and were stained using Prussian blue method to stain iron (a). Pictures were taken at 60× magnification (bar = 50 µm). T24 ($p < 0.001$), MDA-MB-231 ($p < 0.001$) and HT1080 ($p < 0.001$) cells internalized PVA-coated MNPs significantly more than BxPC3, HT29 and LS174T cells (b). No significant difference between the uptake of PVA-coated MNPs by BxPC3, HT29 and LS174T cells. The uptake values are normalized to initial iron concentration of cells. *** $p \leq 0.001$. Bars represent mean ± standard deviation of 12 parallels from two experiments.

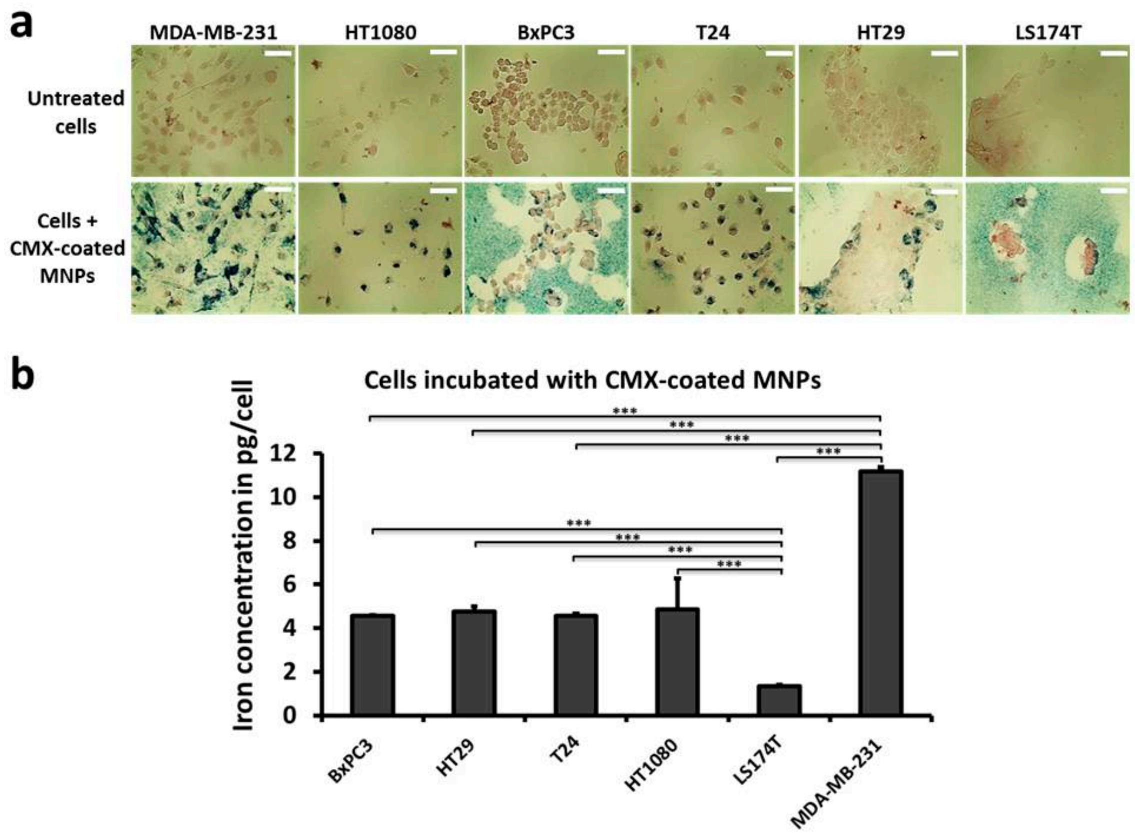

Figure S3. Internalization of CMX-coated MNPs in six different cell lines. Microscopic images of MDA-MB-231, HT1080, BxPC-3, T24, HT29 and LS174T cells internalizing (medium clustering) CMX-coated MNPs (**a**) and the quantified uptake amount of CMX-coated MNPs measured by AAS (**b**). Cells were incubated for 24 h with 100 μg Fe/mL CMX-coated MNPs and subsequently stained by the Prussian blue to visualize iron (a). Images were taken at 60× magnification (bar = 50 μm). To substantiate the uptake level of the medium clustering CMX-coated MNPs in different cell lines, quantification of the iron concentration within the cells was performed after incubation with CMX-coated MNPs. In line with the microscopic results, LS174T showed significantly lower amount of CMX-coated MNPs-based iron than the other cell lines ($p < 0.001$). Whereas the HT29, T24, BxPC3 and HT1080 cells have nearly the same levels of uptake, the internalization rate of CMX-coated MNPs by MDA-MB-231 is clearly higher ($p < 0.001$) (b). The uptake values are normalized to initial iron concentration of cells. *** $p \leq 0.001$. Bars represent mean ± standard deviation of 12 parallels from two experiments.
